# Postoperative Complications and Risk Factors in Skin Tumor Excision With Simple Suturing Under Local Anesthesia: A Multicenter Retrospective Study in Japan

**DOI:** 10.1111/1346-8138.17958

**Published:** 2025-09-15

**Authors:** Kasumi Kato, Hiroshi Kato, Tetsuya Magara, Akimichi Morita

**Affiliations:** ^1^ Department of Geriatric and Environmental Dermatology Nagoya City University Graduate School of Medical Sciences Nagoya Japan

**Keywords:** intraoperative antibiotics, local anesthesia, postoperative complications, risk factors, skin tumor excision

## Abstract

Skin tumor excision under local anesthesia with simple suturing is widely performed in outpatient dermatological practice. Although generally considered safe, postoperative complications such as wound dehiscence, infection, and contact dermatitis can negatively impact patients' quality of life and healthcare resources. However, limited data are available on the complication rates and associated risk factors in procedures without reconstructive techniques. We retrospectively analyzed 1291 patients who underwent local excision and simple closure of skin tumors at three institutions in Japan between January 2021 and November 2024. Complications were defined as adverse events requiring medical intervention within 30 days postoperatively. Receiver operating characteristic curves were used to determine the cutoff values for age, tumor size, operative time, and surgeon experience. Logistic regression analysis was performed to identify independent risk factors. A total of 168 complications occurred in 164 patients (12.7%). The most common symptoms were contact dermatitis (3.7%), wound dehiscence (3.3%), and surgical site infection (2.9%). Multivariate analysis revealed tumor size ≥ 10 mm (odds ratio [OR] 1.52, *p* = 0.040), operative time ≥ 38 min (OR 1.84, *p* = 0.012), and surgeon experience ≥ 10 years (OR 1.64, *p* = 0.013) as significant risk factors. In contrast, intraoperative antibiotic use significantly reduced complication risks (OR 0.29, *p* < 0.001), especially the risk of surgical site infections (OR 0.32, *p* = 0.024). No serious or life‐threatening events occurred. This study identified key predictors of postoperative complications in skin tumor surgery performed under local anesthesia. Larger tumors, longer operative times, and more experienced surgeons were associated with a higher risk, possibly because of the procedural complexity. Prophylactic intraoperative antibiotics effectively reduce infections and should be considered in high‐risk cases. These findings support evidence‐based risk assessment and perioperative management of dermatological surgery.

## 1 Introduction

Skin tumors are a group of diseases commonly encountered in daily practice and include a spectrum of pathologies ranging from benign skin tumors to cutaneous malignancies, such as basal cell carcinoma, squamous carcinoma, and malignant melanoma [1]. Surgical excision is the standard of care for these tumors, and excision under local anesthesia and simple suturing is widely performed, especially in outpatient settings [2]. Advances in dermatologic surgical techniques and the improved safety of local anesthetics have allowed day‐surgery surgical treatment, thereby reducing the physical and economic burden on patients [3].

Nonetheless, various complications have been reported after skin tumor excision, including postoperative bleeding, infection, suture failure, scar contracture, wound opening, and recurrence. These complications not only affect the quality of life (QOL) of patients but may also lead to increased medical resources owing to the need for re‐examination and additional treatment [4]. In addition, patients with underlying conditions such as diabetes may be at even greater risk of complications due to impaired wound healing and delayed immune response [5].

The surgical treatment of cutaneous malignancies has been described, and the complication rates of Mohs micrographic surgery have been analyzed. However, general studies focusing on simple excision and suturing under local anesthesia are limited, and insufficient evidence is available regarding the true incidence of complications and risk factors, especially for standard procedures performed in routine practice. This may be partly due to the clinical background, such as the variability of procedures in individual cases and the fact that minor complications are not often reported in medical records [6].

Furthermore, although multiple factors such as patient background (age, sex, underlying disease) and tumor characteristics (size, location, benign or malignant type) may be involved in the occurrence of complications, few reports have comprehensively analyzed these factors. The technical skills of surgeons and facility responsiveness also have a significant impact on postoperative outcomes, but a quantitative assessment of these factors is needed.

During outpatient treatment, dermatologists must be able to make prompt diagnostic and therapeutic decisions regarding neoplastic lesions and provide safe and reliable treatments. Therefore, identifying the incidence of postoperative complications and their risk factors after excisional surgery is crucial to improving the quality of preoperative informed consent and developing an appropriate postoperative follow‐up plan. In particular, the accumulation and sharing of generalizable data will help to equalize the quality of medical care, especially in the current situation where non‐attending dermatologists increasingly perform surgical procedures for skin tumors in their daily practice.

In this study, we retrospectively analyzed cases of skin tumors (including benign and malignant tumors) excised and sutured under local anesthesia at Nagoya City University Hospital, Seirei Hospital, and Mie North Medical Center, Inabe General Hospital, and aimed to clarify the incidence of postoperative complications and factors related to these complications. In particular, we hoped to clarify which backgrounds and lesions are at risk for simple procedures commonly used in daily practice and to gain knowledge that will contribute to risk assessment and complication prevention measures in future practice.

## 2 Methods

This study was approved by the Ethics Committee of Nagoya City University (approval number: 60‐24‐0162).

We retrospectively collected information from 1532 patients who visited the Department of Dermatology at Nagoya City University Hospital, Seirei Hospital, and Mie North Medical Center, Inabe General Hospital between January 2021 and November 2024. The patients underwent excision and simple suturing under local anesthesia for skin tumors or malignant skin tumors. Of the 1532 patients, 241 were excluded due to insufficient data in their medical records; therefore, 1291 patients were included in the final analysis.

### 2.1 Surgical Procedures

For benign skin tumors, a skin incision was made 1 mm from the tumor margin, and the tumor was excised from the shallow fat layer. For benign subcutaneous tumors, such as calcifying epitheliomas and epidermal cysts, a skin incision was made at the spindle, including the area of skin surface degeneration, and the tumor was excised at the tumor margin. For malignant cutaneous tumors, a safety margin of 2–20 mm was used based on the type of cancer and the expected thickness of the tumor. A skin incision was made at the spindle, and the tumor was resected in the deep fat layer. For subcutaneous malignant skin tumors, a skin incision was made at the spindle with a safety margin of 10 mm from the site of tumor contact, and the tumor was excised from the fascia. Patients with subcutaneous malignant tumors who underwent combined fascial resection were excluded. All skin defects were sutured with a single or two layers of dissolving sutures under the skin, and the surface was sutured and fixed with a non‐dissolving suture. The wound was kept closed for 24 h and was allowed to be washed in the shower the next day. A gauze dressing was applied after washing. The sutures were removed 1–2 weeks later.

### 2.2 Antibiotic Administration

Intraoperative antibiotics were primarily CEZ 1 g in most cases, but 2 g was administered in cases exceeding 80 kg. Additionally, 1 g of CMZ was used for surgeries involving the perineal region. For patients with allergies to certain cephalosporin antibiotics, 1 g of FOM or 600 mg of CLDM was administered. Postoperative antibiotics were administered orally in all cases. Some antibiotics used included CCL 750 mg/day, AMPC/CVA 750 mg/day, and CFPN‐PI 300 mg/day. The administration period was 2–4 days in most cases.

### 2.3 Definition of Complications

Complications were defined as adverse events requiring medical intervention, such as hospital visits, medications, and procedures, and were selected when they occurred within 30 days of surgery. Surgical complications were graded using the Clavien–Dindo classification [7].

### 2.4 Statistical Analysis

Statistical analyses were performed using the Bell Curve for Excel (Social Survey Research Information Co.) and GraphPad Prism version 9.00 for Windows (GraphPad Software, La Jolla, California, USA, www.graphpad.com). Major surgical complications were identified through data analysis, and the receiver operating characteristic (ROC) curve was used to assess the cutoff values for each complication in terms of tumor size, operation time, and years of surgical experience. Items previously reported for each complication and suggested to be related were selected as explanatory variables, and logistic regression analysis was employed to evaluate the clinical findings contributing to postoperative complications [4, 8].

## 3 Results

### 3.1 Patient Characteristics

A total of 324 patients (164 men and 160 women) had malignant skin tumors, and 917 patients (504 men and 413 women) had benign skin tumors. The mean age of patients with malignant tumors was 74.5 years, whereas that of patients with benign tumors was 52.5 years (Mann–Whitney *U* test, *p* < 0.01). The proportion of subcutaneous tumors was significantly lower in the malignant tumor group than in the benign tumor group (Fisher's direct probability test, *p* < 0.01) (Table 1).

**TABLE 1:** The analyzed cases were classified into malignant and benign tumors, and their respective profiles were examined.

Characteristics	Malignant	Benign	
Sex			
Male	164	504	
Female	160	413	
Age			
Average (years)	74.5	52.5	**
Range	22–100	7–96	
Parts			
Body	52	294	
Extremities	81	238	
Face and head	160	326	
Genital	31	109	
Grade of physician			
Average (years)	8.5	8.6	
Range	3 ~ 20	1–38	
Operating time			
Average (min)	33.8	30.5	
Range	9–129	3–107	
Depth			
Cutaneous	305	402	
Subcutaneous	21	563	**
Size			
Average (mm)	14.6	20	
Range	1–120	1–200	

*Note:* Statistically significant differences were identified between benign and malignant tumors in age (Mann–Whitney *U* test, *p* < 0.01) and percentage of subcutaneous tumors (Fisher's direct probability test, *p* < 0.01), respectively. The significance of ** value is p  <  0.01.

### 3.2 Complications and Rates

A total of 168 complications (13%, Table 2) were observed. Complications occurred in 164 patients (12.7%) when duplicates were considered. The most common complication was contact dermatitis caused by tape or antiseptics in 3.7% of the cases. Other complications included open wounds (3.3%), surgical site infection (SSI) (2.9%), numbness or discomfort (1.2%), postoperative bleeding or hematoma (0.9%), severe pain (0.5%), hyperventilation or dysphoria during surgery (0.2%), hives (0.2%), eyelid retraction (0.1%), burns with energy devices (0.1%), and fever (0.1%). No statistically significant difference was observed in the incidence of complications between the malignant and benign tumor groups. No life‐threatening or serious complications requiring hospitalization or intensive care occurred.

**TABLE 2:** The analyzed cases were classified into malignant and benign tumors, and a list of complications was prepared for each.

Complications	All (*n* = 1292)	%	Malignant (*n* = 324)	%	Benign (*n* = 968)	%
Contact dermatitis	48	3.7	9	2.8	39	4.0
II (Medication indicated except for antiemetics, antipyretics, analgesics)	46		8		38	
I (deviation from normal postoperative course)	2		1		1	
Surgical wound opened	43	3.3	13	4.0	30	3.1
IIIa (medical intervention under local anesthesia)	2		0		2	
II (medical management)	34		9		25	
I (clinical observation only)	7		4		3	
Surgical site infection	37	2.9	5	1.5	32	3.3
II (medical management)	34		5		29	
I (clinical observation or diagnostic evaluation only)	3		0		3	
Numbness, discomfort	16	1.2	4	1.2	12	1.2
II (medical management)	2		2		0	
I (clinical observation or diagnostic evaluation only)	14		2		12	
Bleed, hematoma	11	0.9	4	1.2	7	0.7
IIIa (surgical hemostasis under local anesthesia or endoscopic and radiological intervention hemostasis)	6		3		3	
I (controllable with compression only)	5		1		4	
Severe pain	6	0.5	2	0.6	4	0.4
I (clinical observation only; intervention not indicated except for NSAIDs)	6		2		4	
Hyperventilation, moodiness	2	0.2	0	0.0	2	0.2
I (deviation from normal postoperative course)	2		0		2	
Ulticaria	2	0.2	1	0.3	1	0.1
II (medication indicated except for antiemetics, antipyretics, analgesics)	2		1		1	
Entropium ciliarum	1	0.1	0	0.0	1	0.1
I (deviation from normal postoperative course)	1		0		1	
Burn	1	0.1	0	0.0	1	0.1
II (medication indicated except for antiemetics, antipyretics, analgesics)	1		0		1	
Fever up	1	0.1	0	0.0	1	0.1
II (medication indicated except for antiemetics, antipyretics, analgesics)	1		0		1	
All	168	13	38		130	

*Note:* The most common complication was contact dermatitis, followed by wound dehiscence and surgical site infection.

### 3.3 Determination of Cutoff Values With ROC Curves

First, the cutoff values for all types of complications were determined based on the age, tumor size, operative time, and years of experience of the primary surgeon (or the attending surgeon, if one was assigned). The cutoff for age was 45 years, with an FPF of 0.2689, TPF of 0.3232, and OR of 1.2985 for individuals over 45 years old. The cutoff for tumor size was 10 mm in diameter, yielding an FPF of 0.6850, a TPF of 0.7256, and an OR of 1.2160 for tumors ≥ 10 mm. The cutoff for operative time was set at 38 min, with an FPF of 0.2680, a TPF of 0.3902, and an OR of 1.7483 for 38 min or more. The cutoff for years of experience of the primary surgeon was 10 years, with an FPF of 0.3008, a TPF of 0.4817, and an OR of 2.1604 for 10 years or more.

Next, for each complication, we analyzed the cutoff values for the parameters suggested to be associated in previous reports using ROC curves. The cutoff age for contact dermatitis was 52 years, with an FPF of 0.6106, TPF of 0.7083, and an OR of 1.5487 for individuals aged 52 years or older. Next, the cutoff for tumor size at wound opening was 10 mm in the long diameter, with FPF at 0.5408, TPF at 0.5349, and an OR of 1.1281 for tumors ≥ 10 mm. The cutoff for operative time was 26 min, with an FPF of 0.4407 and TPF of 0.5116 for procedures longer than 26 min and an OR of 1.3295. The cutoff for years of experience of the primary surgeon was 7 years, with FPF at 0.3357, TPF at 0.4651, and an OR of 1.7205 for more than 7 years.

The cutoff for operative time for infection was 19 min, with FPF 0.7895, TPF 0.9730, and an OR of 9.6000 for operative times greater than 19 min. The cutoff for tumor size was 17 mm, with an FPF of 0.3246, a TPF of 0.5135, and an OR of 2.1967 for tumor sizes greater than 17 mm. Regarding the years of experience of the primary surgeon, the cutoff was 11 years, with an FPF of 0.2974, TPF of 0.4865, and OR of 2.2376 for 11 years or more. The cutoff for tumor size related to bleeding was 26 mm, with an FPF of 0.1781, TPF of 0.3636, and OR of 2.6366.

### 3.4 Multivariate Analysis of Each Complication Using Logistic Regression

**FIGURE 1:**
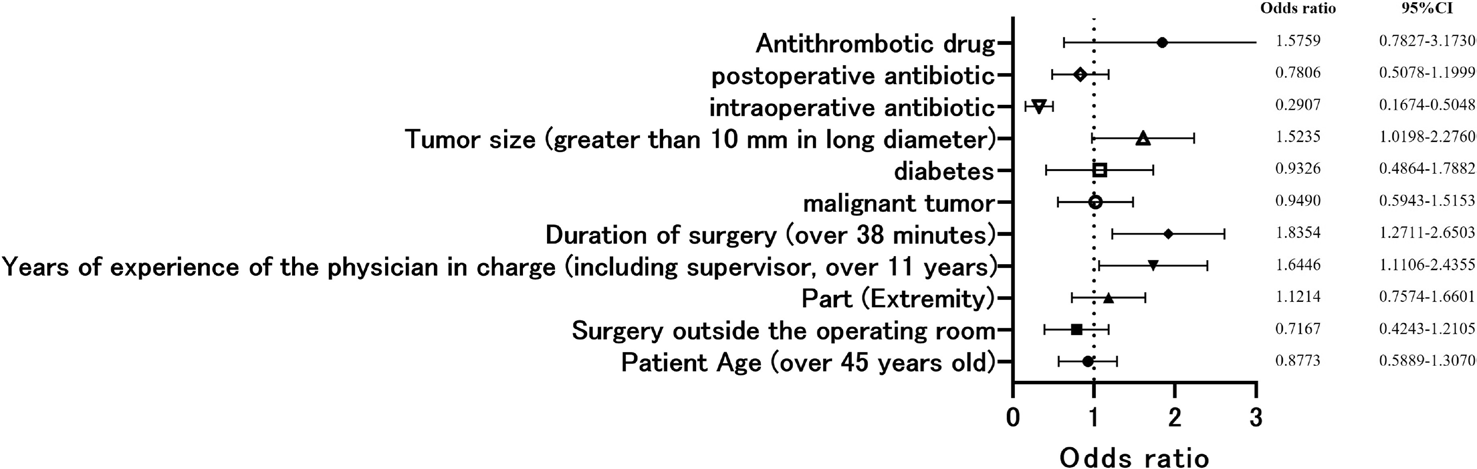
All complications were used as objective variables, and explanatory variables included age, location of surgery (operating room or otherwise), surgical site, years of experience of the primary surgeon, duration of surgery, whether malignant tumor or not, presence of diabetes, tumor size, intraoperative antibiotics administered, postoperative antibiotics administered, and antithrombotic drugs. The risk of all complications increased with the surgeon's years of experience (OR, 1.6446; *p* = 0.013), with the duration of surgery (OR, 1.8354; *p* = 0.012), and with larger tumor size (OR, 1.5235; *p* = 0.040). Characteristically, intraoperative antibiotics reduced the risk of complications (OR, 0.2907; *p* = 0.00001), but postoperative prophylactic medications were not associated with postoperative complications.

Logistic regression was performed using the cutoff values obtained above. The results of the logistic regression analysis with all complications as objective variables are summarized in Figure 1. The risk of all complications was not associated with the patient's age, the setting in which the surgery was performed (operating room or not), site, whether the patient had a malignancy, the presence or absence of diabetes, or the presence or absence of antithrombotic drugs.

In contrast, the risk of complications increased with surgeons' years of experience (OR, 1.6446; *p* = 0.013), duration of surgery (OR, 1.8354; *p* = 0.012), and larger tumor size (OR, 1.5235; *p* = 0.040). However, intraoperative antibiotics reduced the risk of complications (OR, 0.2907; *p* = 0.00001). Postoperative prophylactic medications were not associated with postoperative complications (Figure 1).

Logistic regression was performed on the individual items suggested to be associated with the risk of complications derived from previous reports, and no statistically significant risk factors were found for contact dermatitis or postoperative bleeding. No association was found between bleeding and the presence or absence of antithrombotic drugs or platelet count.

**FIGURE 2:**
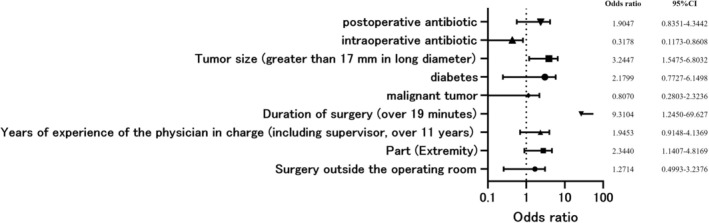
Surgical site infection was the objective variable, and the following explanatory variables were used: Location of surgery (operating room or not), surgical site, years of experience of the primary surgeon, operation time, malignancy or not, presence of diabetes, tumor size, presence of intraoperative antibiotics, and presence of postoperative antibiotics. Results showed that site (extremity) (OR, 2.3440, *p* = 0.020), operative time (OR, 9.3104; *p* = 0.03), and tumor size (OR, 3.2447; *p* = 0.0018) increased the risk of As with all complications, intraoperative antibiotics reduced the risk of postoperative infection (OR, 0.3178; *p* = 0.024) and postoperative antibiotics showed no association and postoperative antibiotics showed no association.

Postoperative wound infection was not associated with the setting in which the surgery was performed (operating room or non‐operating room), the surgeon's years of experience, whether the patient had a malignant tumor, or whether the patient had diabetes mellitus. In contrast, site (extremities) (OR, 2.3440; *p* = 0.020), operative time (OR, 9.3104; *p* = 0.03), and tumor size (OR, 3.2447; *p* = 0.0018) increased the risk of postoperative infection. Similar to all complications, intraoperative antibiotics reduced the risk of postoperative infection (OR, 0.3178; *p* = 0.024), whereas postoperative antibiotics showed no association (Figure 2). SSIs occurred in 1.56% of patients who received intraoperative antibiotics and in 5.8% of patients who did not receive intraoperative antibiotics (Figure 2).

**FIGURE 3:**
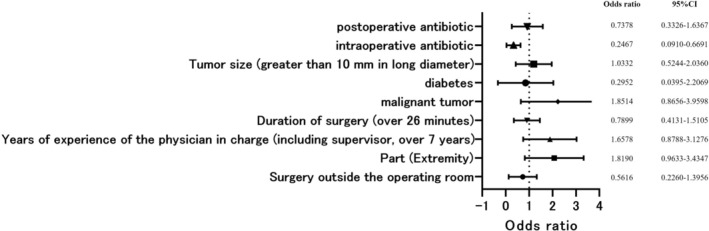
Logistic regression was performed with postoperative wound dehiscence as the objective variable and the following explanatory variables: Location of surgery (operating room or not), surgical site, years of experience of the primary surgeon, operation time, malignancy or not, presence of diabetes, tumor size, presence of intraoperative antibiotics, and presence of postoperative antibiotics. Results showed that intraoperative antibiotics.

Logistic regression analysis of postoperative wound healing (Figure 3) suggested that intraoperative antibiotic therapy may reduce the risk of postoperative wound healing (OR, 0.2467; *p* = 0.0060); however, no other variables were associated with postoperative wound opening (Figure 3).

## 4 Discussion

This study analyzed only cases of skin tumor resection performed under local anesthesia with simple sutures and with no reconstruction methods, such as skin valves or skin grafts. This approach is useful for risk management in clinical practice because it is comparable to office surgery. This study also included the types of complications observed in daily clinical practice, such as contact dermatitis, wound dehiscence, postoperative infection, and bleeding. However, contact dermatitis may also be caused by the tape used to secure gauze dressings. It is also important to note that retrospective studies are subject to subjective interpretation and likely associated with a high degree of error when evaluating the presence of contact dermatitis. This study differs from previous reports in that it provides a comprehensive analysis of the complications that require treatment.

Among the multivariate analyses in this study, the results of the analysis of SSI are noteworthy. In our study, the rate of infection was 2.9%. Schlager et al. performed a meta‐analysis of wound infections after skin surgery and reported that the SSI ranged from 0.96% to 8.70%. This variation was expected because they included several dermatological surgical procedures (e.g., skin grafts and skin valves) [9]. In our study, the infection rate in the group that received intraoperative antibiotics was 1.56%, whereas that in the group without intraoperative antibiotics was 5.80%, suggesting that intraoperative antibiotics may reduce the risk of site infection in a multivariate analysis. Liu et al. analyzed the risk factors of postoperative infection in patients who did not use antibiotics. The infection rate was 4.0%, and the risk of infection was high for ear surgery (OR, 6.03) and surgery involving a large defect area (OR, 1.08/cm^2^) [10]. Kulichová et al. also performed a retrospective analysis of SSI incidence and risk factors for dermatological surgery [11]. The results showed that the incidence of SSI was 1.9%, and high‐risk sites included the head, neck, and extremities. They reported that antibiotics may be unnecessary for low‐risk surgeries; however, their prophylactic use is recommended for high‐risk sites. Our study also suggests that the risk of infection was higher in high‐risk sites, such as the extremities, and that tumor size was also a risk factor for infection. Intraoperative antimicrobial therapy reduced the risk of wound opening postoperatively. Therefore, prophylactic antibiotics are recommended for procedures that require a prolonged operative time, such as with large tumors located in a high‐risk area. The fact that intraoperative prophylactic antibiotics are more effective than postoperative antibiotics is important from the perspective of an antibiotic stewardship program.

Brewer et al. compared the rate of SSIs in patients who used sterile gloves with those who used nonsterile gloves. They found no differences in the rate of postoperative SSIs between the nonsterile glove group (2.1%) and the sterile glove group (2.0%). Although sterile gloves were used in all cases, no differences were observed in the incidence of complications such as infection between the groups that underwent surgery in a non‐operating room environment (e.g., an outpatient clinic or hospital ward) and the group that underwent surgery in the operating room, suggesting that a simple sterile environment can be used in dermatologic surgery [12].

Moreover, the finding that increased length of surgeon experience was associated with an increased risk of complications may seem contradictory, but this could be due to the possibility that high‐risk cases and difficult procedures were performed by experienced surgeons or that the primary surgeon was younger in cases where the attending physician intervened. Subgroup analysis by years of surgeon experience and various parameters revealed no significant differences in surgical site or tumor size. However, statistically significant differences were observed in the presence of diabetes and the use of immunosuppressants. Patients with diabetes or those who were taking immunosuppressants were more likely to be treated by experienced surgeons (*p* < 0.05, Mann–Whitney *U* test) (Figure S1a,b). In fact, in a study by Kaya Erdogan et al. that examined the association between complications in dermatologic and cosmetic surgery and years of surgeon experience among 240 dermatologists, the complication rate was statistically higher among specialists with more than 15 years of experience [13], which is similar to our findings. More detailed studies are needed in the future, including the degree of involvement of supervising physicians.

Postoperative bleeding is an important complication in dermatologic surgery. Bonadurer et al. reported that postoperative bleeding occurred in 3.5% of patients on continuous antithrombotic therapy when dermatologic surgery was performed. In the same report, 2 of 59 patients who discontinued antithrombotic agents had major thromboembolism; therefore, the decision to treat with antithrombotic agents should be made after careful consideration of the risks of surgery and the severity of bleeding complications [14]. We reviewed 1004 cases of complications of punch biopsy for skin diseases and investigated the complications and risk factors. The results showed that the most common complication was postoperative bleeding, with a frequency of 0.9% [15]. The risk of bleeding was listed as sites other than the trunk (OR, 4.600) and a low platelet count (OR, 2.82). No association with bleeding was observed for antithrombotic drugs. The results of our study were similar, suggesting that the risk of bleeding is low in punch biopsies and small operations that can be simply sutured so that antithrombotic drugs can be continued.

Based on the above, we propose the following: if the tumor is larger than 10 mm and located in the extremities or other parts of the body, and if the operation requires more than 20 min, it is recommended that intraoperative antimicrobial agents be administered and that the patient be fully informed before the operation.

This was a retrospective observational study with certain limitations. Some statistical results had wide 95% confidence intervals. For these, we agree that the cutoff value derived from our dataset may be subject to potential overfitting, and the generalizability to other clinical populations is limited. Therefore, the present findings should be interpreted as hypothesis‐generating and require validation in larger, prospective cohorts. In clinical practice, this parameter should be considered as one of several factors that may inform decision‐making, rather than a stand‐alone determinant. For instance, the underreporting of minor complications and omissions in the medical records may have affected the risk assessment. In addition, the surgeon's skill, details of postoperative care, and variations in technique between the centers were not quantitatively determined. In this study, cases in which antibiotics were administered during surgery at the attending physician's discretion were separated from cases in which antibiotics were administered postoperatively. Because there were no fixed criteria, the possibility of bias cannot be ruled out. In the future, prospective observational or multicenter studies are warranted.

## Ethics Statement

This study was approved by the Ethics Committee of Nagoya City University (approval number: 60‐24‐0162).

## Conflicts of Interest

The authors declare no conflicts of interest.

## Supporting information


**Figure S1:** (a) Relationship between surgeon experience and presence of diabetes. Surgeons had more experience with diabetic patients than with nondiabetic patients. Relationship between surgeon experience and use of immunosuppressants. Surgeons had more experience with patients receiving immunosuppressants than with those who were not. Surgeons had more experience with patients receiving immunosuppressants than with those who were not (*p* < 0.05, Mann–Whitney *U* test).

## Data Availability

All authors have full access to all data in this study.

## References

[1] S. K. T. Que , F. O. Zwald , and C. D. Schmults , “Cutaneous Squamous Cell Carcinoma: Incidence, Risk Factors, Diagnosis, and Staging,” Journal of the American Academy of Dermatology 78, no. 2 (2018): 237–247, 10.1016/j.clindermatol.2004.06.013. 29332704

[2] H. Kato , S. Kano , M. Yoshimitsu , et al., “Comparative Analysis of One‐Step and Two‐Step Full Thickness Skin Grafting and Secondary Intention Healing for Skin Defects After Surgical Management of Plantar Malignant Melanoma,” Journal of Dermatology 51, no. 12 (2024): 1641–1645, 10.1111/1346-8138.17398. 39229708

[3] A. J. Dixon , M. P. Dixon , D. A. Askew , and D. Wilkinson , “Prospective Study of Wound Infections in Dermatologic Surgery in the Absence of Prophylactic Antibiotics,” Dermatologic Surgery 32, no. 6 (2006): 819–826, discussion 826, 10.1111/j.1524-4725.2006.32167.x. 16792648

[4] M. Alam , O. Ibrahim , M. Nodzenski , et al., “Adverse Events Associated With Mohs Micrographic Surgery: Multicenter Prospective Cohort Study of 20,821 Cases at 23 Centers,” JAMA Dermatology 149, no. 12 (2013): 1378–1385, 10.1001/jamadermatol.2013.6255. 24080866

[5] T. Isei , M. Abe , R. Ikegami , et al., “Wound, Pressure Ulcer, and Burn Guidelines – 3: Guidelines for the Diagnosis and Treatment of Diabetic Ulcers and Gangrene, Second Edition,” Journal of Dermatology (2025), 10.1111/1346-8138.17697. 40292848

[6] C. F. Heal , P. G. Buettner , and H. Drobetz , “Risk Factors for Surgical Site Infection After Dermatological Surgery,” International Journal of Dermatology 51, no. 7 (2012): 796–803, 10.1111/j.1365-4632.2011.05189.x. 22715823

[7] H. Katayama , Y. Kurokawa , K. Nakamura , et al., “Extended Clavien‐Dindo Classification of Surgical Complications: Japan Clinical Oncology Group Postoperative Complications Criteria,” Surgery Today 46, no. 6 (2016): 668–685, 10.1007/s00595-015-1236-x. 26289837 PMC4848327

[8] J. M. Amici , A. M. Rogues , A. Lasheras , et al., “A Prospective Study of the Incidence of Complications Associated With Dermatological Surgery,” British Journal of Dermatology 153, no. 6 (2005): 967–971, 10.1111/j.1365-2133.2005.06861.x. 16225607

[9] J. G. Schlager , D. Hartman , J. Wallmichrath , et al., “Patient‐Dependent Risk Factors for Wound Infection After Skin Surgery: A Systematic Review and Meta‐Analysis,” International Wound Journal 19 (2022): 1748–1757, 10.1111/iwj.13780. 35229471 PMC9615300

[10] X. Liu , M. Sprengers , P. J. Nelemans , K. Mosterd , and N. W. J. Kelleners‐Smeets , “Risk Factors for Surgical Site Infections in Dermatological Surgery,” Acta Dermato‐Venereologica 98, no. 2 (2018): 246–250, 10.2340/00015555-2844. 29136259

[11] D. Kulichová , T. Geimer , M. Mühlstädt , T. Ruzicka , and C. Kunte , “Surgical Site Infections in Skin Surgery: A Single Center Experience,” Journal of Dermatology 40, no. 10 (2013): 779–785, 10.1111/1346-8138.12255. 23961937

[12] J. D. Brewer , A. B. Gonzalez , C. L. Baum , et al., “Comparison of Sterile vs Nonsterile Gloves in Cutaneous Surgery and Common Outpatient Dental Procedures: A Systematic Review and Meta‐Analysis,” JAMA Dermatology 152, no. 9 (2016): 1008–1014, 10.1001/jamadermatol.2016.1965. 27487033

[13] H. Kaya Erdogan , M. S. Tekin , E. Agaoglu , et al., “Emergency Complications During Dermatological, Surgical, or Cosmetic Procedures: A Cross‐Sectional Study Among Dermatologists,” Journal of Cosmetic Dermatology 23, no. 12 (2024): 4102–4109, 10.1111/jocd.16479. 39032133 PMC11626380

[14] G. F. Bonadurer , A. P. Langeveld , S. C. Lalla , et al., “Hemorrhagic Complications of Cutaneous Surgery for Patients Taking Antithrombotic Therapy: A Systematic Review and Meta‐Analysis,” Archives of Dermatological Research 314, no. 6 (2022): 533–540, 10.1007/s00403-021-02250-x. 34132885

[15] Y. Yasui , H. Kato , T. Oda , M. Nakamura , and A. Morita , “Complications and Risk Factors of Punch Biopsy: A Retrospective Large‐Scale Study,” Journal of Dermatology 50, no. 1 (2023): 98–101, 10.1111/1346-8138.16585. 36151785

