# Supplementary figures and images for: Postoperative Complications and Risk Factors in Skin Tumor Excision With Simple Suturing Under Local Anesthesia: A Multicenter Retrospective Study in Japan
Source: J Dermatol. 2025 Sep 15;52(12):1803–10. doi: 10.1111/1346-8138.17958 (PMC12698892; doi:10.1111/1346-8138.17958)

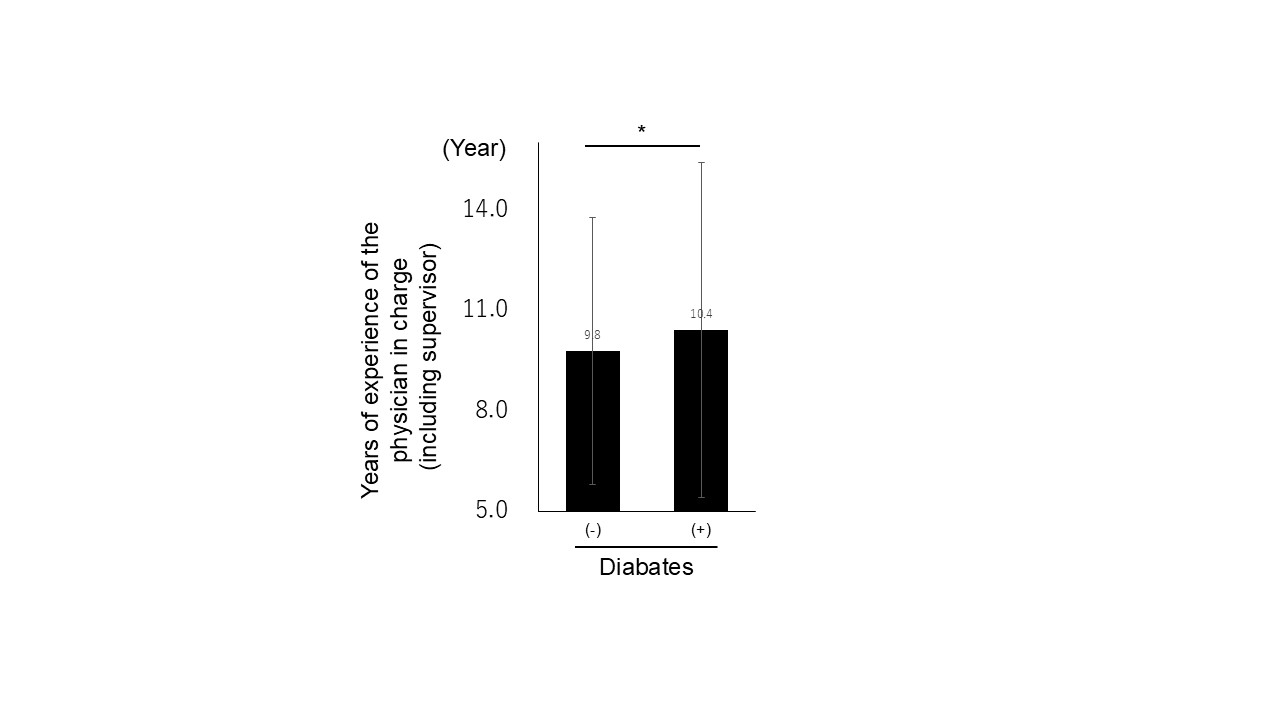

Supplement: Supplementary file 1 — Figure S1: (a) Relationship between surgeon experience and presence of diabetes. Surgeons had more experience with diabetic patients than with nondiabetic patients. Relationship between surgeon experience and use of immunosuppressants. Surgeons had more experience with patients receiving immunosuppressants than with those who were not. Surgeons had more experience with patients receiving immunosuppressants than with those who were not (p < 0.05, Mann–Whitney U test). [file JDE-52-1803-s001.zip › jde17958-sup-0001-Supinfo_1.JPG]

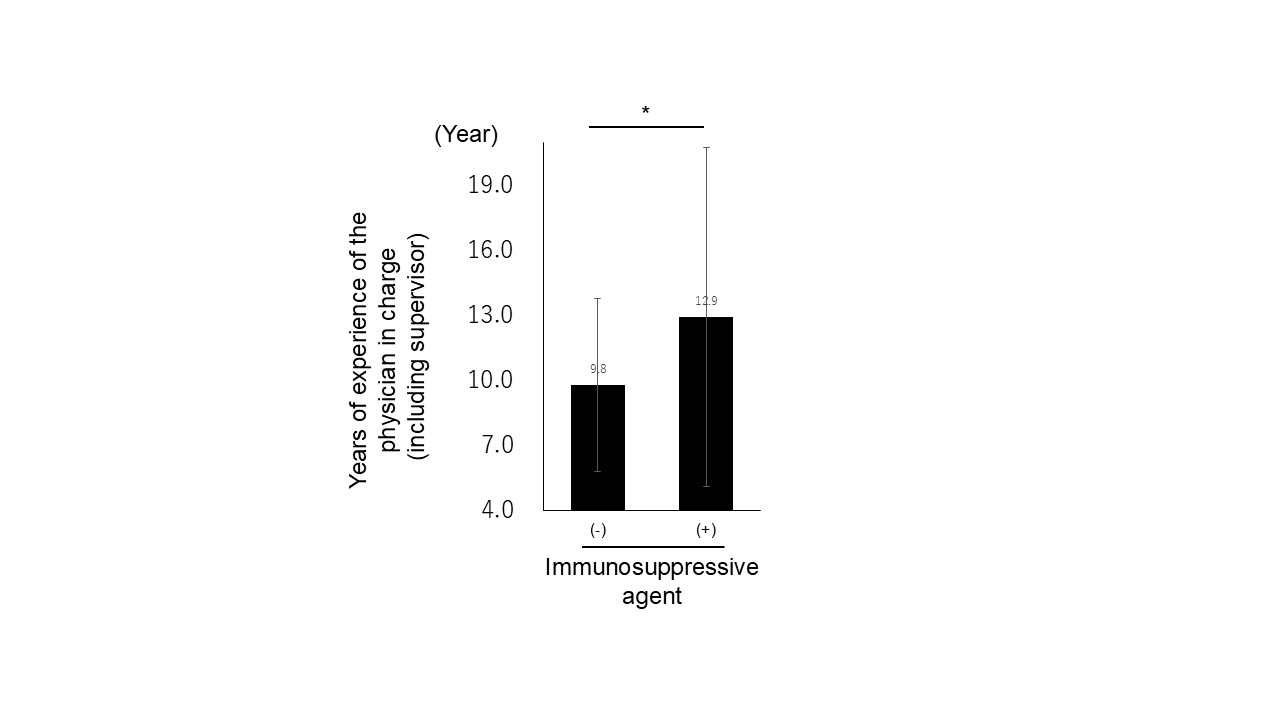

Supplement: Supplementary file 1 — Figure S1: (a) Relationship between surgeon experience and presence of diabetes. Surgeons had more experience with diabetic patients than with nondiabetic patients. Relationship between surgeon experience and use of immunosuppressants. Surgeons had more experience with patients receiving immunosuppressants than with those who were not. Surgeons had more experience with patients receiving immunosuppressants than with those who were not (p < 0.05, Mann–Whitney U test). [file JDE-52-1803-s001.zip › jde17958-sup-0002-Supinfo_2.JPG]
